# Future directions for patient engagement in research: a participatory workshop with Canadian patient partners and academic researchers

**DOI:** 10.1186/s12961-024-01106-w

**Published:** 2024-02-14

**Authors:** Anna Maria Chudyk, Roger Stoddard, Todd A. Duhamel, Patient Engagement in Research Partners, Brenda Andreas, Maureen C. Ashe, Jennifer Daly-Cyr, Sarah Elliott, Audrey L’Esperance, Melissa Park, Monica Parry, Martine Puts, Mariann Rich, Bryn Robinson, Donna Rubenstein, Sherald Sanchez, Kurt Schreiner, Lesley Singer-Norris, Kathy Smith, Gillian Strudwick, Karine Toupin-April, Suzanne Vercauteren, Katie Wadden, Annette S. H. Schultz

**Affiliations:** 1 College of Pharmacy, Rady Faculty of Health Sciences, University of Manitoba, CR3024-369 Tache Avenue, Winnipeg, MB R2H 2A6 Canada; 2 Horizon Health Network, 80 Woodbridge Street, Fredericton, NB E3B 4R3 Canada; 3 Faculty of Kinesiology and Recreation Management, 212 Active Living Centre, Winnipeg, MB R3T 2N2 Canada; 4 Institute of Cardiovascular Sciences, St. Boniface General Hospital-Albrechtsen Research Centre, 351 Tache Ave, Winnipeg, MB R2H 2A6 Canada; 5 College of Nursing, Rady Faculty of Health Sciences, University of Manitoba, CR3022-369 Tache Avenue, Winnipeg, MB R2H 2A6 Canada

**Keywords:** Patient engagement, Patient engagement in research, Patient-oriented research, Participatory workshop, Patient and public involvement, Patient involvement, Multi-stakeholder engagement, Capacity building, Participatory process

## Abstract

**Background:**

Patient engagement in research (also commonly referred to as patient or patient and public involvement in research) strives to transform health research wherein patients (including caregivers and the public) are regularly and actively engaged as multidisciplinary research team members (i.e. patient partners) working jointly towards improved health outcomes and an enhanced healthcare system. To support its mindful evolution into a staple of health research, this participatory study aimed to identify future directions for Canadian patient engagement in research and discusses its findings in the context of the international literature.

**Methods:**

The study met its aim through a multi-meeting pan-Canadian virtual workshop. Participants (*n* = 30) included Strategy for Patient-Oriented Research-funded academic researchers and patient partners identified through a publicly available database, personal and professional networks and social media. All spoke English, could access the workshop virtually, and provided written informed consent. The workshop was composed of four, 1.5–3-h virtual meetings wherein participants discussed the current and preferred future states of Canadian patient engagement in research. Workshop discussions (i.e. data) were video and audio recorded. Themes were generated through an iterative process of inductive thematic analysis that occurred concurrently with the multi-week workshop.

**Results:**

Our participatory and iterative process identified 10 targetable areas of focus for the future of Canadian patient engagement in research. Five were categorized as system-level (systemic integration; academic culture; engagement networks; funding models; compensation models), one as researcher-level (engagement processes), and four crossed both levels (awareness; diversity and recruitment; training, tools and education; evaluation and impact). System level targetable areas called for reshaping the patient engagement ecosystem to create a legitimized and supportive space for patient engagement to be a staple component of a learning health system. Researcher level targetable areas called for academic researchers and patient partners to collaboratively generate evidence and apply knowledge to inform values and behaviours necessary to foster and sustain supportive health research spaces that are accessible to all.

**Conclusions:**

Future directions for Canadian patient engagement in research span 10 interconnected targetable areas that require strong leadership and joint action between patient partners, academic researchers, and health and research institutions if patient engagement is to become a ubiquitous component of a learning health system.

**Supplementary Information:**

The online version contains supplementary material available at 10.1186/s12961-024-01106-w.

## Background

The process of joint knowledge production, in which research is conducted with instead of on participants, has a rich history in social sciences research [1]. Notably, in the 1940s, Kurt Lewin (working in the United Kingdom and United States) developed a theory of action research that followed a cycle of continuous inquiry, action and evaluation and was conducted with marginalized groups for social action and not just scholarly outputs [1–3]. In the 1970s, Paulo Freire published the *Pedagogy of the Oppressed* on the basis of his study of oppressive educational environments in Brazil, critically examining concepts central to the co-production of knowledge between researchers and those traditionally considered research participants, including thematic investigation (solving one’s own problems by critically reflecting on them), emancipation (collective action towards systemic transformation) and the nature of power dynamics [3–5]. Although both scholars’ work focussed on empowering *communities* to bring about change, they are widely considered to be the ‘northern’ and ‘southern’ originators of participatory action research, which is rooted in the belief that those most impacted by research should be involved from the beginning to the end of the research process, including helping to develop the research questions, plan and conduct the study, analyse findings and decide the products and actions most useful to effecting change [1, 3, 6]. With time, the participatory action approach to research has permeated across the different branches of scientific inquiry and diverged in terms of its underlying terminology and the individuals and groups involved in the co-production of the research [7].

Broadly speaking, patient engagement in research refers to a spectrum of research that is co-produced with patients and other members of the public (including caregivers, family members, patient representatives and/or advocates) through a wide range of activities in which patients have varying degrees of influence on study decision-making [8, 9]. Patients and other members of the public who are engaged in this co-production are commonly referred to as “patient partners” [10]. As the exact terms and definitions of this research approach vary geographically (e.g. patient and public involvement, stakeholder engagement) [11, 12], we have chosen to use the terms “patient engagement in research” and “patient partners” throughout this paper for consistency [10]. As a form of participatory action research, patient engagement in research has been defined as “the active, meaningful, and collaborative interaction between patients and researchers across all stages of the research process, where **research decision-making** is guided by patients’ contributions as partners, recognizing their specific experiences, values, and expertise” [12]. Established in 1996 and 2010, The National Institute for Health and Care Research (NIHR) in the United Kingdom and Patient-Centered Outcomes Research Institute (PCORI) in the United States are widely considered to be global institutional leaders of patient engagement in research, significantly shaping current approaches through their institutional models and championing and support of patient engagement in research [13–16].

In 2011, the Canadian Institutes of Health Research (CIHR) established the Strategy for Patient-Oriented Research (SPOR) as a targeted approach to bridging knowledge gaps within the evidence-to-practice continuum through POR, a continuum of patient engagement research that focusses on patient- and public-identified priorities and outcomes [10, 17]. SPOR acts as a catalyst for POR in several ways, including by (a) establishing provincial, territorial and national-level centres of expertise (e.g. SPOR SUPPORT Units, SPOR Evidence Alliance, SPOR Canadian Data Platform) aimed at supporting and building capacity to conduct POR and to use the results from this research to inform learning health systems, (b) directly funding POR through two main mechanisms, SPOR Networks (pan-Canadian collaborative research networks that focus on specific health areas identified as priorities in multiple provinces and territories) and the SPOR Innovative Clinical Trials Initiative (funding opportunities aimed at expanding the spectrum of innovative clinical trials research), and (c) identifying and supporting synergy between patients, caregivers, researchers, healthcare providers and policy-makers [18]. Since SPOR’s inception, an increasing number of Canadian studies have engaged patient partners in the co-production of research, as evidenced by the proliferation of SPOR research networks and the studies affiliated with them, as well as CIHR grant calls that require patient engagement. However, if SPOR and CIHR are to become among the established institutional leaders of patient engagement in research, and patient engagement is truly to become a staple of Canadian health research, more work must be done to co-produce a mutual vision for its future directions in Canada.

To our knowledge, only three studies have directly investigated future directions for patient engagement in research in Canada, all from the perspectives of academic researchers (specifically trainees and early career researchers) [19–21]. Given its participatory action roots, this lack of representation of the patient perspective in the published research is an important gap that paints an incomplete picture. Looking beyond Canada, future directions for patient engagement in research have been recently examined in the United States and United Kingdom from the perspectives of academic researchers and patient partners [14, 22]. While these reports can provide some guidance and relevant comparisons, they do not replace context-specific data. Furthermore, our reflections on potential future directions have pushed us to consider the understudied role of the system and the academic researcher–patient partner ecosystem in shaping the patient engagement in research climate [23–26]. Specifically, while health and biomedical research commonly focusses on the individual and modifiable behaviours, within patient engagement a more complete picture only begins to emerge when considering the individual within the context of the system [23–26].

## Methods

### Aim

In an effort to support the continued evolution of patient engagement in research in Canada, we carried out an exploratory pan-Canadian multi-session workshop that engaged SPOR-funded academic researchers and patient partners in dialogue about the current and preferred future states of patient engagement in research. Our over-arching research question was, “What is the preferred future state of patient engagement in research in Canada in the next 5–10 years?” Our study aim was to identify future directions for Canadian patient engagement in research. We locate our findings at the levels of the researcher (i.e., patient partner or academic researcher) and system so as to guide considerations of, and maximize the applicability of our findings to, both researchers and the systems they function in.

### Setting and study design

This participatory design study was based out of University of Manitoba affiliated St. Boniface Research Centre (Winnipeg, Canada), and conducted virtually with SPOR-funded academic researchers and patient partners from across Canada. It was the final component of a three-part mixed-methods project that aimed to describe the enactment of patient engagement in SPOR-funded projects through a cross-sectional survey [27] and interviews with patient partners [28] and identify future directions for the field (current study). Ethics approval was obtained from the Education Nursing Research Ethics Board at the University of Manitoba (certificate number E2019:082(HS23180)).

### Participants and recruitment

Workshop participants were patient partners and academic researchers (i.e. principal/co-applicants, research or engagement support staff) on SPOR-funded projects (2014–2019) who could communicate in English, provide informed consent and participate in the workshop virtually. Study eligibility was not affected by whether patient partners and academic researchers engaged in previous research together. A multi-modal recruitment strategy utilized personal (i.e. our project’s prior study participants interested in future research opportunities) and publicly available (i.e. CIHR’s funding decisions) databases, personal and professional networks and social media (i.e. Twitter). The lead author (AMC) compiled a list of potential participants through the databases and research team networks. She then emailed invitations to potential participants chosen at random from the combined databases and all those nominated through research team networks. The invitations included a study overview, consent form and request to contact her if interested in learning more about the workshop. The email also welcomed the recipient to share the invitation with SPOR-funded academic researcher and patient partner colleagues. Two reminder emails were sent approximately 2 weeks apart. We also distributed the same study information as a recruitment poster that summarized the study to our networks for wider distribution, which included their Twitter accounts. Recruitment took place between 30 November 2021 and 1 February 2022, and ended when we reached our target sample size of 15 patient partners and 15 academic researchers. This sample size was chosen on the basis of group dynamics (including group sizes conducive to meaningful small-group and full-group discussions) and feasibility (e.g. funding, scheduling, staffing). All participants provided written informed consent prior to study participation. Patient partner participants were offered a $250 honorarium and academic researcher participants were offered a $100 honorarium (electronic gift card or cheque).

### Data collection

**Fig. 1:**
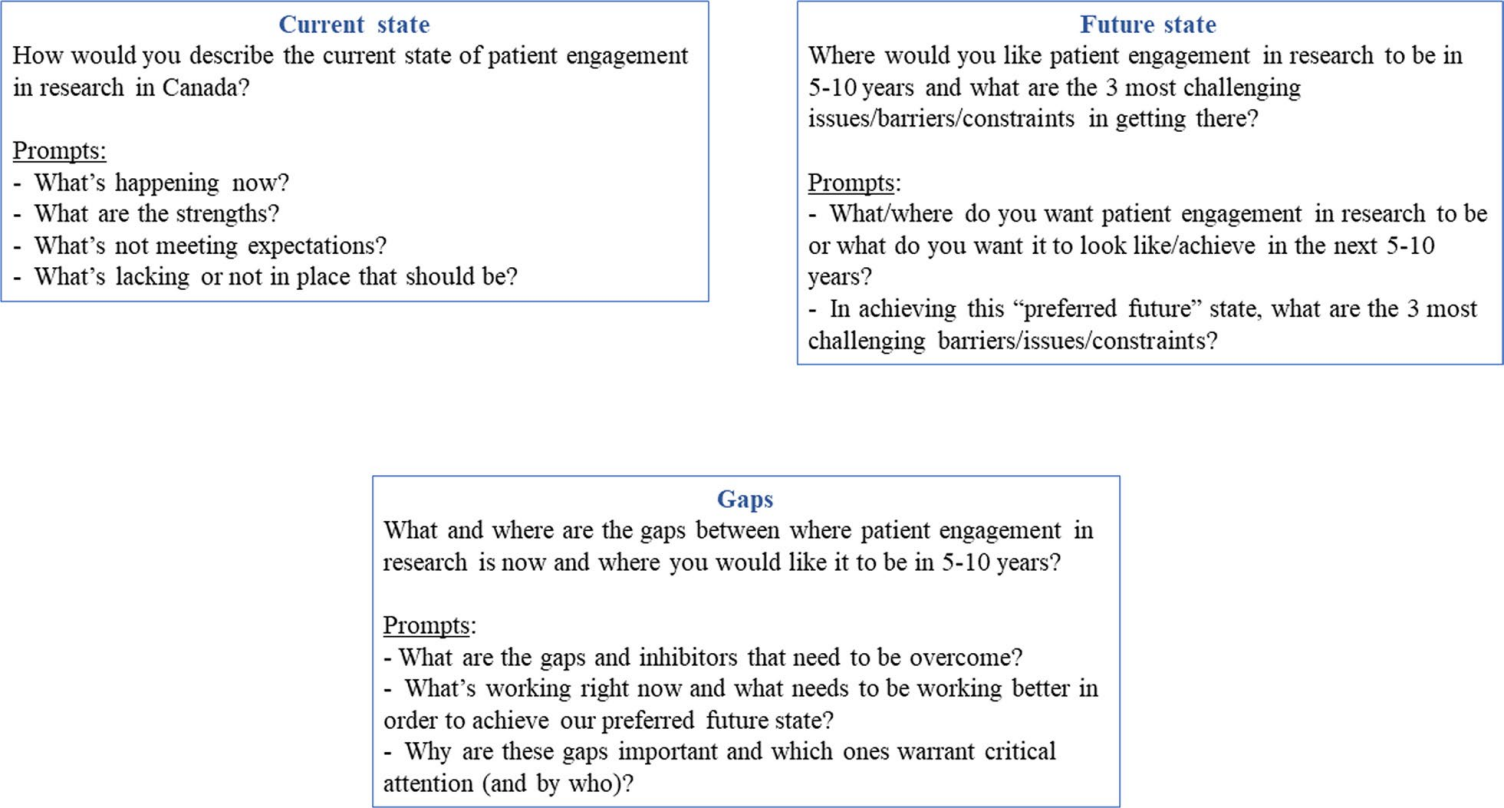
Workshop overview

Four 1.5–3-h virtual meetings were held between 15 February and 24 March 2022. Session agendas are found in Additional file 1. In the first meeting, patient partners and academic researchers met separately with their respective stakeholder groups to discuss the current and preferred future states of Canadian patient engagement in research and the gaps that lie between them (Fig. 1). Afterwards, the study team created and shared a summary document that outlined the similarities and differences between each stakeholder group’s discussions, which were further explored and member-checked with each stakeholder group separately during the second meeting. In the third meeting, participants came together to discuss similar study questions through small group activities that blended participants from both stakeholder groups. By first having the opportunity to explore and establish their own stakeholder group’s perspectives on the study questions, and then learn about the perspectives of the other stakeholder group through the summary report, we aimed to balance power dynamics between academic researchers and patient partners and create an environment in which both stakeholder groups would come into the third session with a better understanding of their and others’ perspectives as well as increased comfort in discussing them. Consequently, this approach also intended to create the space for more synergy to occur than if the meetings blended the stakeholder groups from the start. Finally, the last meeting was aimed at discussing key study findings and knowledge mobilization. Participants who were unable to attend a workshop meeting had the option of submitting their responses to the discussion prompts to the lead author (AMC) for sharing at the meeting and/or incorporation into the analysis. All four meetings were co-led by the first author (RS) and study’s patient partner (RS) and small group discussions were supported by trained facilitators that represented the patient partner and academic researcher perspectives. This design was purposefully chosen so as to further contribute to the balancing of power dynamics between academic researchers and patient partners through tangible demonstrations of shared leadership and valuing of both perspectives. We collected participants’ sociodemographic information through a self-report questionnaire completed before the first meeting. Workshop proceedings were documented through video recordings and written notes made by the meeting facilitators. The experience-based co-design approach [9] informed the workshop’s multi-sessions structure, and the strategic planning process influenced the questions posed at each session.

### Patient and stakeholder engagement

This study was conceptualized, planned and conducted in collaboration with a patient partner (RS), who was a co-investigator and co-author. This patient partner was identified through his previous patient engagement work with the research team [29, 30], including helping develop the grant that supported the underlying three-part research project. As a collaborator, (RS) contributed equally to the decisions that were made about the study [8, 9], and chose how and when he wanted to contribute across the study’s research cycle through conversations with the study’s lead author (AMC) at their regularly scheduled (~ bi-weekly) check-in meetings. The study also consulted [8, 9] patient partner and academic researcher participants through member-checking. That is, participants helped shape and ensure the credibility of data analysis and synthesis by providing written or verbal feedback on summary reports created after the first and last meeting. They were also invited to stay engaged during knowledge dissemination by providing critical feedback on this article prior to peer-review submission. Those who reviewed the manuscript were offered co-authorship under a group name.

### Analysis

Workshop discussions (i.e. data) were video and audio recorded. Themes were generated through an iterative process of inductive thematic analysis [31, 32] that occurred concurrently with the multi-week workshop. Workshop participants provided input on the analysis (i.e. member-checking) between the workshop sessions, and this promoted a richer and deeper understanding of the data. Specifically, after the first meeting, the first author (AMC) engaged in data triangulation by reviewing written participant responses (for those who could not attend a meeting) and facilitator notes with the workshop recordings to ensure the notes’ accuracy, support clarification of any missing components and become familiar with the data. She then coded participants’ responses for each question individually, noting unique and overlapping concepts for each stakeholder group. These preliminary codes were then reviewed and grouped into themes by her. The initial themes were reviewed and revised by the study’s patient partner (RS) and senior author (ASHS) and summarized in a document sent to workshop participants for written feedback and discussion during the study’s second meeting. The themes were then further revised by the study’s co-authors (only) after meetings 2 and 3, following a similar process to the one outlined above. Prior to meeting 4, the lead author (AMC) constructed a thematic map to support an understanding of the relationships between themes [33] using MindManager 2020 software (Corel Corporation, USA). Specifically, the thematic map visualized targetable areas that could bridge identified gaps between the current and preferred future states of patient engagement in research in Canada. After the thematic map was reviewed by the research team, it further informed the writing of an overall findings summary report, which was sent out for member-checking by workshop participants and resulted in completed data analysis. Sociodemographic data are presented using summary statistics generated using IBM SPSS Statistics 27 (IBM, USA).

## Results

We invited 14 patient partners and 118 academic researchers identified through personal and public databases and 14 patient partners and 37 academic researchers identified through personal and professional networks to participate in the study. Of these, a total of 15 patient partners and 15 academic researchers participated in the workshop. All patient partners attended meetings 1, 2, and 4, and 14 patient partners attended meeting 3. A total of 14 academic researchers attended meeting 1, 13 academic researchers attended meeting 2, 14 academic researchers attended meeting 3, and 13 academic researchers attended meeting 4. Workshop participants’ sociodemographic characteristics are presented in Table 1.

**Table 1:** Sociodemographic characteristics of study participants (*n* = 29)^a^

	Patient partner, *n* (%)	Academic researcher, *n* (%)
Age, years	63.8 (5.3)^b^	43 (11.3)^b,c^
Gender	13 (93%) Women 1 (7%) Man	14 (93%) Women 1 (7%) Prefer not to say
Ethnicity (self-identified categories)	8 (58%) White/Caucasian 2 (14%) European Canadian 1 (7%) Black Canadian 1 (7%) Canadian 1 (7%) Ashkenazi Jewish 1 (7%) Afro-Caribbean	5 (32%) White/Caucasian 3 (20%) European 2 (13%) French Canadian 1 (7%) Chinese Canadian 1 (7%) Japanese Korean 1 (7%) Filipino 1 (7%) South Asian 1 (7%) Prefer not to say
Place of residence (province/territory)	7 (50%) Ontario 2 (14%) Quebec 2 (14%) Nova Scotia 1 (7%) British Columbia 1 (7%) Alberta 1 (7%) Saskatchewan	7 (46%) Ontario 3 (20%) Quebec 2 (13%) British Columbia 1 (7%) Alberta 1 (7%) New Brunswick 1 (7%) Newfoundland
Highest level of education completed	1 (7%) High school 5 (35%) Bachelor’s degree 8 (58%) Master’s degree	N/A
Primary community represented	6 (43%) Patient 1 (7%) Caregiver 7 (50%) Both patient and caregiver	N/A
Position	N/A	7 (46%) Professor (assistant/associate/full) 2 (13%) Research centre director 2 (13%) PhD student 1 (7%) Research program manager 1 (7%) Engagement manager 1 (7%) Research associate 1 (7%) Postdoctoral fellow
Years of experience being engaged/engaging patient partners	6.5 (3.3)^b^	6.8 (3.8)^b^
Years of experience being engaged/engaging patient partners in SPOR-funded opportunities	4.4 (1.8)^b^	4.7 (2.2)^b^
Types of SPOR-funded studies and/or SPOR-funded opportunities worked on	13 (93%) Research study 5 (36%) SPOR network 1 (7%) SPOR SUPPORT unit 2 (14%) SPOR	14 (93%) Research study 4 (27%) SPOR network 3 (20%) SPOR SUPPORT unit

^a^*n* = 1 patient partner did not complete the sociodemographic survey; ^b^mean (standard deviation); ^c^*n* = 1 missing; SPOR, Strategy for Patient-Oriented Research

**Fig. 2:**
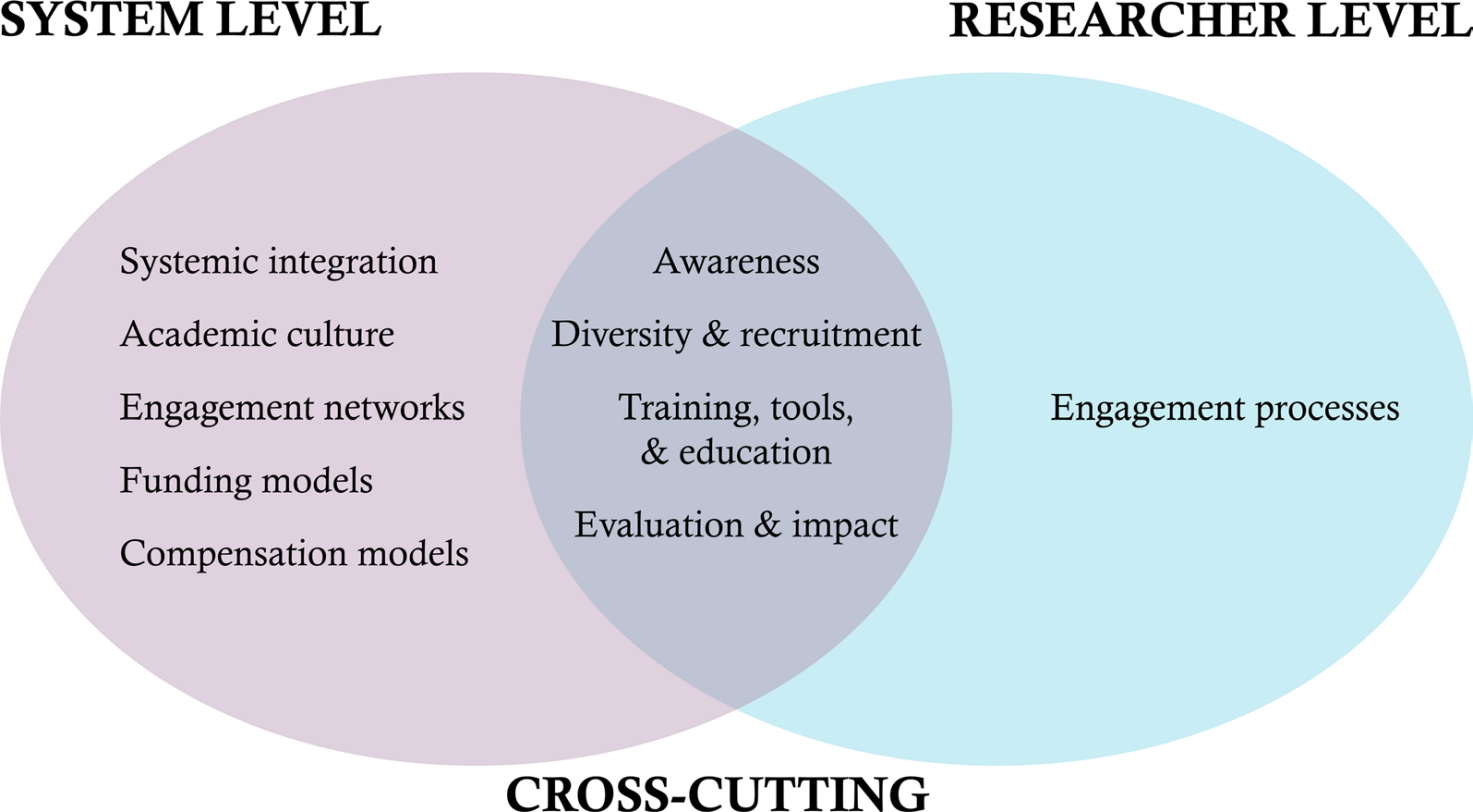
Conceptual summary of study findings

Our participatory and iterative process identified 10 targetable areas of focus (Fig. 2) for the future directions of Canadian patient engagement in research. Five of the targetable areas were located at the system level (i.e. systemic integration; academic culture; engagement networks; funding models; compensation models), one at the researcher (individual/interpersonal level, i.e. engagement processes), and four crossed both levels (i.e. awareness; diversity and recruitment; training, tools and education; evaluation and impact). We next describe the characteristics of the preferred future state by targetable area. Each description begins with a brief overview of the perceived current state, as characterized by workshop participants, to provide some context within which to consider the preferred future state.

### Targetable areas that reside at the system level

#### Systemic integration

*Current state:* Patient engagement in research is not systemically integrated within and across the Canadian healthcare and research systems. The healthcare system is relatively unaware of patient partners’ value and importance, and research findings do not consistently drive patient care.

*Future state:* Patient engagement in research is systemic, meaning it permeates the structures of the Canadian research and healthcare systems. This future state is supported through:

- *Designated patient partner positions*. It is standard practice for research and healthcare organizations to have designated positions for patient partners within their leadership and strategic governance organizational structures, as well as other organizational levels specific to areas of research and care. These compensated positions help ensure that patient partner interests are represented at the systems level, by patient partners who have the necessary resources and supports (e.g. financial, time) for the positions, and support patient partner involvement in setting research and healthcare agendas and priorities that lead to innovative funded projects and patient-centred healthcare reform.
- *Embedment of SPOR within the pillars of CIHR*. SPOR (and patient engagement in general) is embedded within and across all of the pillars of CIHR (i.e. biomedical, clinical, health systems services and population health) with structural supports enabling action and opportunities, thereby helping ensure that engagement is the status quo from benchside to bedside.
- *Bi-directional relationship between research and healthcare*. The Canadian research and healthcare systems are intricately connected to form a learning health system, wherein patient-identified priorities, outcomes and perspectives drive research, and patient-engaged research informs healthcare delivery and decisions.

#### Academic culture

*Current state:* Current research pedagogy often prioritizes empirical knowledge over experiential knowledge, which can lead to academic researchers not valuing the patient perspective and the contributions it brings to research, and institutional cultures not valuing faculty research that foregrounds patient perspectives and the contributions they bring to research. Academic culture, and especially career requirements, often pose disincentives for patient engagement in research, especially among tenure-track faculty.

*Future state:* The values and structures that underlie academic culture have evolved to better support patient engagement in research, including its systemic integration into research and health systems. These changes include shifts in:

- *Principles and values*. Academic culture respects and values the contributions and experiences of every research partner, including those with lived experience.
- *Metrics*. Academic career metrics support and acknowledge the time and financial resources required to meaningfully engage patient partners, including engaging in innovative research approaches that require sharing power and the spotlight and the production of alternate knowledge translation products that are often of greater interest to patient partners and more accessible to the public.
- *Policies*. Policies support and encourage the active participation of patient partners in graduate student committees, tenure and promotion reviews and capacity building (e.g. training, education), knowledge mobilization (e.g. conferences) and peer-review processes, all of which contribute to and maintain the shift in values and metrics of academic culture.

#### Engagement networks

*Current state:* There is a lack of organizational forums (e.g. communities of practice, professional networks) that foster a culture of engagement within and among the patient engagement community. This has a negative impact on patient partner recruitment/retention, diversity and inclusion and overall uptake of patient engagement in research. There is also a lack of clarity about existing SPOR networks and other infrastructure to support engagement (e.g. what exists, how to access, roles and responsibilities), which deters from full utilization of existing capacity to support patient engagement in research.

*Future state:* Local and national patient engagement networks and conferences exist to bring people together, offer opportunities for organic development of research partnerships and deliberate grafting of perspectives less often heard in research and advance the awareness and culture of patient engagement within both research and public communities. Specifically, this includes:

- *Organizational forums*. There is universal clarity about existing SPOR infrastructure (e.g. networks and opportunities) that maximize the effectiveness and utilization of these infrastructures. Patient partner societies, communities of practice and leadership groups are well established, and unified and interconnected engagement networks exist nationally and across disease processes. Taken together, these organizational forums offer centralized, easily findable and streamlined support, training and networking opportunities for all research partners, enhance patient partner recruitment and public awareness of patient engagement in research and are supported through evolved funding models.
- *Networking and gathering opportunities*. Patient-engagement-focussed peer support, mentorship and gathering opportunities are abundant for patient partners, academic researchers, students and the general public. This could, for example, include establishing an annual knowledge mobilization forum (such as the SPOR Summit) spearheaded by a national platform or established network, refocussing and repositioning existing knowledge mobilization forums with patient partners at the forefront and/or other regularly occurring events such as workshops or webinars.

#### Funding models

*Current state:* Patient engagement in research is heavily dependent on CIHR funding. As described in Additional file 1: Table S1, several issues with the current funding system negatively impact patient engagement in research, including funding allocations, grant application and review processes, and lack of post-grant accountability that evaluates whether and how funds are spent on patient engagement activities.

*Future state:* Funding opportunities and review processes are in place that enable patient engagement to traverse all aspects of Canadian health research and help ensure that patient and caregiver priorities are driving research. Accountability mechanisms are built into grant funding to support the integrity of engagement processes and build up the evidence base that underlies patient engagement in research. Specifically, this includes:

- *Diverse funding opportunities*. There is a widespread availability of diverse funding sources (e.g. increased investments from universities, healthcare systems and use of crowd-sourcing) to support patient engagement in research, including more funding being available for early career researchers, trainees and small teams to engage patient partners during idea generation and grant development stages, and for patient partner-led projects.
- *Designated patient partner positions*. Patient partners regularly have opportunities to take on meaningful roles (e.g. ones that directly influence priorities, scores and outcomes) in funding allocation and grant peer-review processes (and are well supported to do so through compensation, training, etc.), thereby helping ensure that research focusses on patient and caregiver priorities and outcomes and is driven by patient and caregiver perspectives.
- *Accountability mechanisms*. Research teams are required to demonstrate a base understanding of patient engagement when applying for grants, as supported by informational supports (e.g. modules such as those available through CIHR for sex- and gender-based analyses). Awarded grants require reporting of indicators that assess adherence to proposed engagement plans and formal evaluation of engagement processes. These accountability mechanisms help enhance proposed engagement plans, promote more meaningful engagement and support the commonplace evaluation of engagement.

#### Compensation models

*Current state:* Patient partner compensation is often inconsistent and unequal across projects and institutions and not comprehensively considered within grant budgets, especially among researchers new to patient engagement. Institutional structures result in frequent delays in time to be paid for research contributions, and inflexible compensation policies disproportionately affect patient partners on fixed incomes and other unique populations and act as a barrier to increasing diversity among patient partners.

*Future state:* Compensation practices have evolved to diminish power imbalances between academic researchers and patient partners, contribute to patient partners feeling valued and ensure equal opportunity for engagement across diverse groups. These changes include increased opportunities for patient partners to be hired full time (e.g. onto studies, by institutions). Key mechanisms that contribute to the achieving and maintaining this future state include:

- *Standards and guidelines*. National institutions and/or funding agencies have set consistent and equitable compensation standards and guidelines, which are flexible enough to allow for consideration of unique needs (e.g. patient partners on fixed incomes), and are adopted by universities, research bodies and researchers. Consequently, patient partners are consistently and equitably compensated across Canada and engagement activities are comprehensively represented in grant budgets (across the research cycle from idea generation to knowledge dissemination).
- *Streamlined and flexible policies*. Institutions have resolved university and funding agency policies and structures that complicate, limit and slow down how/when patient partners are compensated and that lack the flexibility necessary to address the unique needs of all patient partners. They also have agreements in place that meet the requirements of funders, and patient engagement standards and guidelines.

### Targetable areas that reside at both the system and researcher levels

#### Awareness

*Current state:* Although interest is growing, there is still an overall lack of awareness of patient engagement in research and its benefits among the general public and research communities. This may be due to a lack of knowledge translation aimed at helping the public and researchers better understand the approach, its value and process of involvement; infrequent sharing of study information and findings specifically targeted at the public; and a lack of opportunities for academic researchers and patient partners to talk about the engagement process and their experiences.

*Future state:* There is widespread awareness of patient engagement in research (including its benefits, preferred approaches and how to get involved in it). This enables engagement to become an actual cornerstone of Canadian health research and supports an increased prevalence of engagement among academic researchers and the public. Key researcher and system level approaches to achieving and maintaining this state include:

- *Knowledge mobilization*. Research findings (especially of studies that engage patients) are readily accessible by the general public, not just people in the research world.
- *Centralized portal/organization*. There is an established space for current and potential research partners (including the public) to connect, publicize ongoing and completed research (to reduce redundancies and increase awareness) and host conferences and other public events.

#### Diversity and recruitment

*Current state:* There is a lack of diversity among (a) patient partners, (b) fields of research, researcher career stages and team sizes engaging patient partners and (c) the overall number of patient partners (versus other researchers) within research teams. Academic researchers are unsure of where and how to find patient partners, with current patient partners often feeling obliged to partner on multiple projects as a result. The public is unsure of how to partner on research, and opportunities to become a patient partner are largely dependent upon existing relationships.

*Future state:* Patient partners better reflect the socio-demographic makeup and perspectives of all Canadians, including under-served and under-represented communities and those with different experiences and roles (e.g. caregivers) within the healthcare system. Patient partners are regularly being engaged across the fields of research, with researchers at all career stages and in teams composed of multiple patient partners that support each other in navigating the research landscape, which further supports patient engagement becoming ubiquitous to all facets of Canadian research. Key researcher and system level supports that contribute to achieving and maintaining this future state include:

- *Informational supports*. Tools and strategies from a variety of delivery modes (e.g. print, visuals, video) are readily available and utilized to support recruitment and partnership with diverse people onto the research team.
- *Structures*. Traditional and alternative structures exist across levels of stakeholders (e.g. health research funding agencies, government, institutions, projects, clinicians) to support diversity in recruitment, perhaps including a centralized system such as a national registry or other bodies that connect academic researchers, patient partners, members of the public and projects. These structures complement grassroots relationship building, active outreach to communities and supporting the development of local capacity for research engagement within communities (e.g. helping establish a patient partner advisory council within a community organization or patient advocacy group).

#### Training, tools and education

*Current state:* Academic researchers’ and patient partners’ knowledge of patient engagement methods is inconsistent and often lacking – both groups commonly learn about engagement through ‘doing’. Available patient-engagement-related training opportunities and informational supports are limited and not well indexed, and it is primarily up to academic researchers to provide training and education to patient partners. There is a notable lack of opportunities to learn about and gain experience with patient engagement in medical and graduate education programs, which is compounded by structures that do not typically support the presence of patient partners on student advisory committees.

*Future state:* Academic and patient research partners have access to the content knowledge necessary to partner to their full potential, including informational supports that help eliminate key knowledge gaps, presented in Table 2. All trainees have the opportunity and are strongly encouraged to learn about patient engagement approaches through supportive training environments that provide them with regular opportunities to interact with and learn from patient partners early in their careers. This not only increases knowledge of patient engagement in research, but also helps foster an academic culture in which all viewpoints are valued and patient engagement is standard practice. Key mechanisms that support the development and maintenance of this future state include:

- *Centralized and readily accessible informational support*. The information, training and tools required for academic researchers and patient partners to partner to their full potential exist, including consensus statements and best practice guidelines. These are easily and universally accessible across Canada through coordinated portals (e.g. websites) that reduce redundancies and support uptake of high-quality information (such as the ones developed for sex- and gender-based analyses).
- *Integration into higher education curricula*. Dedicated positions (for patient partners and patient-oriented researchers) and championing by leadership/administration has led to patient engagement approaches being integrated into diverse higher education curricula and widespread opportunities for patient partners to sit on graduate committees and interact with trainees (e.g. through courses, mentorship, experiential learning opportunities).

**Table 2:** Topics that require more training, tools and education to support effective patient engagement in research

Key topics presented in alphabetical order	
Advocating for yourself (as a patient partner) and your voice in the room	Equity, diversity and inclusion considerations
Authorship and intellectual property	Establishing and negotiating expectations among research partners
Barriers to engagement experienced by patient partners	Ethics (e.g. when consent/assent is not needed from patient partners)
Budgeting for engagement	Evaluation of engagement processes
Communicating with patient partners and affirming the value they bring	Initiating and maintaining patient partnerships
Compensation (of patient partners)	Patient engagement in the basic sciences
Creating the space for patient partner input	Sharing lived experiences in the research and public setting
Engaging patient partners with diverse backgrounds and viewpoints	The value of patient partners

#### Evaluation and impact

*Current state:* There is a lack of Canadian-specific research and evidence about the benefits, value, outcomes and impacts of patient engagement in research. The available evidence has limited transferability and generalizability across studies. Few validated tools have been developed to adequately evaluate patient engagement in research and few studies evaluate their engagement activities formally (e.g. using tools) or informally (e.g. through check-ins with academic and patient partners), perhaps due to a lack of established evaluation frameworks, indices and metrics that incorporate both patient partner and academic researcher perspectives.

*Future state:* Evaluation and measurement of the processes and impacts of patient engagement have become commonplace. Evaluation tools that fully capture the impact of patient engagement in research are being developed and validated. Evidence to support patient engagement in research is available to guide best practices, justify its cost to funders (including taxpayers), enhance systemic integration, and ensure its long-term sustainability, including establishing patient engagement as best practice in health research. This is achieved through:

- *Indicators and metrics*. There is widespread agreement and recognition of the indicators and metrics – both short term and long term – used to evaluate the outcomes of patient engagement in research and its ‘success’. Innovative and transdisciplinary forms of evaluation that examine both research quality and quantity are utilized in recognition of the fact that standard approaches to health research evaluation may not fully capture the impact of patient engagement in research.
- *Research focussed on the process of engagement*. Research regularly focusses on the process of engagement (e.g. what is optimal engagement, tools to assess engagement, the impact of engagement-related training) to provide better evidence to guide patient engagement throughout research stages, which is especially important when there are limited measures of accountability.
- *Publication guidelines*. Manuscript requirements have evolved to better support the full reporting of engagement methods. Patient engagement reporting guidelines are mandated by journals and utilized by researchers.

### Targetable area that resides at the researcher level

#### Engagement processes

*Current state:* There is growing awareness and understanding that relationships and engagement activities are at the heart of patient engagement in research. However, as outlined in Additional file 1: Tables S2 and S3, many research teams still do not give ample consideration to both. As a result, power imbalances and tokenistic engagement remain common, and patient partners often feel unvalued or unwanted, ultimately diminishing the potential and effectiveness of patient engagement in research.

*Future state:* There is a universal understanding that patient engagement in research is not a one-size-fits-all approach as well as a commitment to maximizing the quality and depth of engagement for each research context. It is standard practice for research teams to carefully consider and address the relational (e.g. emotional intelligence, interpersonal and soft skills, mutual respect) and activity-related (e.g. accountability, mutual understanding of expectations and motivations for engagement) aspects of the engagement process. In particular:

- *Respect and valuing of the patient voice*. Lived experience is considered an essential interdisciplinary lens informing research, and there is widespread recognition that patient partners bring expertise on health conditions and healthcare experiences.
- *Re-distribution of power*. Patient partners are more regularly leading and co-leading studies with the support of academic researchers and restructured funding models.
- *Opportunities to have meaningful impact*. Patient partner engagement regularly begins with priority setting and idea generation and proceeds throughout the research cycle and into knowledge mobilization.
- *Roles and responsibilities*. Patient partners’ roles are negotiated and clearly understood by academic and patient research partners and reinforced through supportive engagement processes.
- *Designated engagement support persons*. Patient engagement liaisons are staple members of research teams, acting as bridges between research partners to address power differentials, support the relational and activity-related aspects of engagement and contribute towards the dynamics conducive to productivity.
- *Minimization of technological barriers to participation*. Strategies are in place to address technology-related inequities at the individual (e.g. internet cards, loaner laptops) and community (e.g. community access hubs) levels. Hybrid engagement models (i.e. combination of in-person and virtual meetings) and necessary supports (e.g. properly equipped meeting rooms, IT support people) are readily available among research teams.

## Discussion

Our study engaged academic researchers and patient partners in a participatory process that culminated in the identification of 10 targetable areas of growth for patient engagement in research in Canada. Taken together, system level targetable areas call for a reshaping of the patient engagement ecosystem to create a legitimate and supportive space for patient engagement to be a staple component of a learning health system that is accessible to all Canadians. Researcher level future directions call for researchers to collaboratively generate evidence and apply knowledge to inform values and behaviours necessary to foster and sustain supportive health research spaces for all Canadians. Each targetable area represents a disconnect between the perceived current and preferred future states of patient engagement in research, and identifies system and researcher level characteristics necessary to establish and maintain an environment of co-production between different stakeholder groups (in this case, patients and academic researchers). In this way, the findings are not only applicable to the Canadian patient engagement in research climate, but can also lend insights into how to better support co-production among different branches of participatory action research and among different Canadian contexts. Specifically, system level targetable areas highlight how structures and policies shape underlying culture and can better support desired opportunities and approaches to research co-production. Researcher level targetable areas stress the importance of generating evidence and raising awareness for approaches to co-production that consider both relational and activity-related aspects and engage stakeholders from priority and idea generation through to knowledge mobilization. The targetable areas are offered as a guide for required changes within the Canadian patient engagement ecosystem, many of which are supported by previously identified barriers to patient engagement in research [14, 34, 35]. A further discussion comparing our study with previous published work within Canada and from leading international patient engagement institutions (i.e. NIHR and PCORI) is provided below. See Additional file 1: Table S4 for a summary of these comparators.

To our knowledge, only three other studies have directly investigated Canadian academic researcher (i.e. trainee and early career) perspectives on future directions for patient engagement and POR [19–21]. As expanded upon in Additional file 1: Table S4, these academic-researcher-focussed publications include a report on the proceedings of the 2017 Knowledge Translation Canada Summer Institute (whose focus was patient-oriented research (POR) and patient engagement in research) [19], a national online survey of pain research trainees [20] and a Delphi survey of a cohort of POR-award recipients within Quebec [21]. Collectively, these studies identified: considerations specific to the knowledge base that underlie patient engagement in research and the building and sustaining of relationships between academic researchers and patient partners [19]; recommendations to improve the implementation of patient engagement in research [20]; and key features of the anticipated future state of POR and early career researchers’ role in supporting POR’s development and implementation [21]. With the exception of systemic integration, all of the targetable areas identified in our current study are supported as future directions for patient engagement in research/POR by the aforementioned Canadian studies, although the specific features of these targetable areas varied between the studies. Further, only our study identified systemic integration as a future direction and investigated perspectives among patient partners and academic researchers at varying career points.

When looking more broadly at the international literature, we could not locate any recent USA-based studies that set out to identify future directions for patient engagement in research from the perspectives of academic researchers or patient partners. Therefore, we turned to the major funder of US patient-engaged research (PCORI) for insights. PCORI is an independent non-profit organization established in 2010 to fund patient-centred comparative clinical effectiveness research. Notably, its governance structure already includes patient partners across different levels of the organization, which could serve as a model for systematically incorporating patient partners within health funding organizations within Canada. As outlined in Table 3, PCORI’s most recent (2022) strategic plan touches upon many of the targetable areas identified through our study. However, its big picture focus is supporting the health of the nation through patient-centred comparative effectiveness research, rather than our narrower focus on supporting the growth of patient engagement in research. Areas of overlap with our study findings include an overarching commitment to equity, diversity and inclusion; a focus on systemic integration towards the development of a learning health system; increasing awareness about the value and outcomes of patient-centred research; development and dissemination of training, tools and education; evaluation and impact; and supporting best practices in engagement processes (including early engagement and across the research cycle) [22]. Although the strategic plan also details funding-related considerations, these focus on identified research areas that support PCORI’s national research priorities. As compared with the findings of this current study, the strategic plan does not explicitly consider systemic integration of patient partners in research and health systems, academic culture, engagement networks, patient partner compensation or recruitment infrastructure. It is possible that these areas are already well reflected within PCORI’s current state, and so not an area of future focus. Regardless, PCORI’s strategic plan offers interesting insights into the future direction of patient engagement in US research, and also reveals differences in focus between institutions and individuals, thereby reaffirming the importance of studies such as ours focussed on developing a holistic vision for future directions and contributing depth to the understanding of identified targetable areas.

In 2018, Staniszewska et al. published a review that reflected upon the progress of patient engagement in research in the NIHR and proposed a vision for 2025 [14]. Unique to the other discussed studies, this review was commissioned at the system level to guide NIHR’s future vision for patient engagement in research, including the cultural and organizational development required to fulfil the vision. Numerous data sources were used to inform this review, including an online survey of a multi-level group of national and international stakeholders (e.g. patients, caregivers, academic researchers, clinicians, patient organizations, charities, policy-makers). As detailed in Additional file 1: Table S4, the review identified key areas of future development for patient engagement, future directions for the design and delivery of patient engagement in the NIHR and key components of an overall vision for the future. Although very similar in its findings, aspects of our study not reflected in NIHR’s future directions included compensation models, professional networks and organizational forums for patient partners and academic researchers, considerations of academic culture and the role of patient engagement in higher education curricula and regular embedment of patient partners in governance positions. This discrepancy in findings could reflect the NIHR’s long-standing commitment and experience with patient engagement in research, respondents feeling as though these areas are already well represented in the perceived current state of patient engagement in the NIHR and/or context-related differences (e.g. infrastructure, organizational or funding structure). Importantly, this study models a novel, national evidence-informed approach towards identifying future directions for patient engagement in Canada.

### Future directions

In considering how our study’s findings can be applied to inform future directions, it is important to acknowledge that SPOR has established a strong foundation for the existence and growth of patient engagement in research in Canada through multi-faceted capacity-building initiatives, including directly funding research, establishing entities that support POR nationally and across Canada’s provinces and territories and through training and career development opportunities that support the creation and application of POR by a wide range of stakeholders [18]. As modelled by the exemplary work of Staniszewska et al. [14], development of a truly national vision for Canadian patient engagement in research requires a strategic approach that incorporates multiple forms of evidence and engages all of the different levels of stakeholders that affect and/or are affected by patient engagement in research. However, studies such as this current one can be used to inform system and researcher level changes that support the evolution of patient engagement into a staple of Canadian research. For example, we have applied this study’s findings towards (a) the co-development and co-conduct of a graduate-level course on approaches to patient engagement in research as a step towards better integrating patient engagement into higher education curricula and (b) developing a podcast (asperusual.substack.com) to help raise awareness for patient engagement-related activities occurring within Canada and to further disseminate this study’s findings within and outside academia. A major support for the widespread application of this study’s findings (and a concerted evolution of patient engagement in general) would be the development of a centralized, universally accessible and well-indexed inventory of available resources and exemplary models of study and system level engagement that are already occurring in Canada, such as those already available through SPOR funded entities (see, for example, [36–39]), and Canadian research groups developing the evidence base that underlies the targetable areas identified through this study (see, for example, [40–43]). Our planned next steps for this current work include the development of such a resource in the context of a study that sets out to co-develop an actionable national research agenda, using the identified future directions as its building blocks. Lastly, given the international similarities and differences in identified future directions for patient engagement in research, the global patient engagement community would benefit from opportunities such as international conferences where researchers could share and discuss current approaches and next steps for patient engagement in research.

### Strengths and limitations

This study has many strengths. Notably, the study was conceptualized, designed and conducted in collaboration with a patient partner (RS), and also offered participants multiple opportunities to shape the analysis, synthesis and write up of the study findings. Further, the study’s data collection activities were mindfully designed to disrupt potential power imbalances traditionally held between patient partners and academic researchers. By taking these steps, our study sought to better ensure that our findings truly reflected the balanced and collective vision of academic researchers and patient partners. Another strength is the inclusion of academic researchers and patient partners from across Canada – as well as in the case of academic researchers, different career points and roles – which resulted in a richness of experiences and perspectives that informed our study findings.

This study also has limitations that warrant mention. Our recruitment approach was not designed to ensure diversity among our study participants, nor did we offer resources to support digitally excluded participants in taking part in the study. Unsurprisingly, it appeared that few participants represented the voices of equity-deserving groups and other groups typically less heard from in research (e.g. youth, patient partners that are male and without post-secondary education). As a result, identified future directions likely do not reflect considerations specific to the under-represented groups of patient partners. Further, we only included academic researchers and patient partners already engaging in SPOR-funded activities. This ensured shared lived experiences relevant to the workshop discussions but omitted the viewpoints of other key stakeholders identified through the study findings, such as the general public, decision-makers within institutions that fund or support patient engagement in research and patient partner networks not funded by SPOR. Relatedly, we chose to limit our sample size to 15 academic researchers and 15 patient partners as this was an exploratory study that required a relatively large time commitment (across multiple sessions) from study participants so as to allow for the disruption of power dynamics between participant stakeholder groups and support conversation depth. Future investigations should purposively recruit a greater range and diversity of perspectives across different types of stakeholder groups and fields of health research, and mindfully ensure that the voices of the minority are not lost among the perspectives of the majority. Lastly, this study was purposely designed to identify the ‘what’ and ‘why’ of where things need to go instead of creating an operational strategy for how to get there. This a planned next step for our research, and an interesting area of future research for system level thinkers interested in further examining how the history of our research structures have shaped the health research environments we live in and how these learnings can best be applied to affect system level changes.

## Conclusions

Future directions for Canadian patient engagement in research span 10 interconnected targetable areas that require strong leadership and joint action between patient partners, academic researchers and health and research institutions if patient engagement is to become a ubiquitous component of a learning health system. Although the majority of the identified targetable areas reside at the system level, it is important to remember the participatory action roots of patient engagement in research and the important roles that the groups of individuals affected by a problem play in effecting change. Thus, it is important to create more space for dialogue to occur through, for example, studies such as this one, to create the critical mass and momentum necessary for changes to the system to occur. Relatedly, information regarding system level changes can also help us to see where we can collaboratively focus our efforts and transform the system together. We all play an important role in shaping the current and preferred future states of patient engagement in research.

### Supplementary Information

**Additional file 1.** Agendas for Meetings 1-4** Table S1.** Summary of issues with current funding models that were identified by study participants. **Table S2.** Summary of relational aspects of the engagement process that that were identified by participants as needing improvement. **Table S3.** Summary of activity-related aspects of the engagement process that were identified by participants as needing improvement. **Table S4.** Overview of key studies examining future directions for patient engagement in research.

## Data Availability

The data that support the findings of this study are available on reasonable request from the corresponding author (Dr. Chudyk). The data are not publicly available due to containing information that could compromise the privacy of research participants.

## Abbreviations

CIHR: Canadian Institutes of Health Research.
NIHR: National Institute for Health and Care Research
PCORI: Patient-Centered Outcomes Research Institute
POR: Patient-oriented research
SPOR: Strategy for patient-oriented research
