# Supplementary material for: Future directions for patient engagement in research: a participatory workshop with Canadian patient partners and academic researchers
Source: Health Res Policy Syst. 2024 Feb 14;22:24. doi: 10.1186/s12961-024-01106-w (PMC10865599; doi:10.1186/s12961-024-01106-w)
Supplement: Supplementary file 1 — Additional file 1. Agendas for Meetings 1-4 Table S1. Summary of issues with current funding models that were identified by study participants. Table S2. Summary of relational aspects of the engagement process that that were identified by participants as needing improvement. Table S3. Summary of activity-related aspects of the engagement process that were identified by participants as needing improvement. Table S4. Overview of key studies examining future directions for patient engagement in research. [file 12961_2024_1106_MOESM1_ESM.docx]

# Additional files

**Additional File 1. Agendas for Meetings 1-4**

**Meeting 1 Agenda**

1. **Welcome, land acknowledgment, and meeting overview.**
2. **Research team introductions.**
3. **Establishing some guidelines for our “virtual” interactions.**
4. **Presentation.**

- Why we’re here, what we’re doing, what we hope to achieve.

1. **Breakout group activity #1**

- ***Question*: *How would you describe the current state of patient engagement in research in Canada?***
  - Prompts (to stimulate discussion): Describe what’s happening now? What are the strengths? What’s not meeting expectations? What’s lacking or not in place that should be?
- We will begin with group members introducing themselves by sharing: what are 1 or 2 important things you want people to know about you OR what are 2 positive things that have happened to you this year?
- We will then discuss the question using a round-robin format (to ensure everyone has an opportunity to share their ideas) and then likely move into a more “open-group” discussion.
- We will spend the last 5-10 minutes asking everyone to share what they think some of the main points from the discussion were (have a pen and paper handy to write them down if possible) so we can report them back to the main group.

1. **Stretch and bio (washroom) break.**
2. **Reporting back from the small groups.**
3. **Breakout group activity #2**

- ***Question*: *Where would you like patient engagement in research to be in 5-10 years and what are the three most challenging issues/barriers/constraints in getting there?***
  - Prompts (to stimulate discussion): What/where do you want patient engagement in research to be or what do you want it to look like/achieve in the next 5-10 years? In achieving this “ideal future” state, what are the three most challenging barriers/issues/constraints?
- We will begin with group members introducing themselves by sharing: what are 1 or 2 important things you want people to know about you OR what are 2 positive things that have happened to you this year?
- We will then discuss the question using a round-robin format (to ensure everyone has an opportunity to share their ideas) and then likely move into a more “open-group” discussion.
- We will spend the last 5-10 minutes asking everyone to share what they think some of the main points from the discussion were (have a pen and paper handy to write them down if possible) so we can report them back to the main group.

1. **Stretch and bio (washroom) break.**
2. **Reporting back from the small groups.**
3. **Big group discussion**

- Bringing it all together**: *What and where are the gaps between where patient engagement in research is now and where you would like it to be in 5-10 years?***

1. **Closing remarks and thank you!**

**Meeting 2 Agenda**

1. **Welcome, land acknowledgment, and meeting overview.**
2. **Research team introductions.**
3. **Reminders.**
4. **Going over our Meeting 1 summary document.**
5. **Wrap-up**

**Meeting 3 Agenda**

1. **Welcome, land acknowledgment, and meeting overview.**
2. **Research team member introductions.**
3. **Re-establishing some guidelines for our “virtual” interactions.**
4. **Presentation.**

- Recap (reminder of our work to date) and what we hope to achieve today.

1. **Breakout group activity #1**

***QUESTION: Keeping in mind that after this meeting, we hope to have a complete picture of what we see as the current and preferred future states of patient engagement in Canada….***

***What information would you like to add, build upon, or highlight in how we have collectively described the current state of patient engagement in research in Canada and what/where we want it to be in the next 5-10 years?***

- Prompts (to stimulate discussion):
- Did any ideas come out of meeting 1 that prompted you to think further about how we characterized the current and/or preferred future states of patient engagement in research in Canada?
- Was anything missing from our previous characterizations that you’d like to add? Are there any ideas you’d like to discuss further?
- Format:
- We will begin with group members introducing themselves by sharing one important thing they’d like people to know about them OR one positive thing that happened to them this year.
- We will then discuss the question using a round-robin format (to ensure everyone has an opportunity to share their ideas) and then likely move into a more “open-group” discussion.
- We will spend the last 5-10 minutes asking everyone to share what they think some of the main points from the discussion were (have a pen and paper handy to write them down if possible) so we can report them back to the main group.

1. **Stretch and bio (washroom) break.**
2. **Reporting back from the small groups.**

**Meeting 4 Agenda**

1. **Welcome, land acknowledgment, and meeting overview.**
2. **Research team member hellos.**
3. **Breakout groups.**

- We will be discussing 4 main questions:

1. What do you believe are the most important messages that you would like to see come out of this work?
2. Who might be interested in our studying findings (and how should we consider getting our work out to them)?
3. What are other potential knowledge translation outputs (other than a manuscript)?
4. What are potential next steps/future directions?
5. **Reporting back.**
6. **Wrap-up and thank-yous.**

**Table S1. Summary of issues with current funding models that were identified by study participants.**

| Aspect | **Key issues** |
| --- | --- |
| Funding allocations | Mostly benefit big teams.  Lack of “seed” funding available to support early engagement (i.e., during idea generation/grant development). |
| Grant application process | Short application deadlines prevent meaningful engagement.  Canadian Common CV and Researchnet requirements pose barriers to patient partners occupying formal roles on grants. |
| Grant review process | Funding criteria do not typically weigh patient engagement processes and evaluation plans.  Requirements for, and evaluations of, patient partner contributions (e.g., letters of support, curriculum vitaes) are unclear and inconsistent.  Involvement of patients in grant reviews (alongside academic researchers) is still uncommon.  When patients are involved in grant reviews, the technical language found in most grants means that they typically need to be highly educated to understand what they are reviewing. |
| Post-grant accountability | Lack of accountability about how/whether funds are spent on engagement.  Lack of funder-mandated evaluation of engagement activities. |

Table S2. Summary of relational aspects of the engagement process that that were identified by participants as needing improvement.

| **Aspect** | **Key issues** |
| --- | --- |
| Emotional intelligence | Characteristics such as empathy, conscientiousness, and self-awareness require increased focus for successful engagement. |
| Interpersonal and soft skills | Not all research teams are mindful of how their interpersonal and soft skills (e.g., active listening, honesty, warmth) contribute to/detract from an environment that is conducive to successful engagement. |
| Mutual respect | Appreciation for the experience and role of patient partners is not yet universal, and research partners need to cultivate mutual respect for everyone’s expertise. There also needs to be an increased understanding that respect is expressed through words and actions like considering patient partner schedules in planning meetings and establishing reasonable time frames for providing input. |

Table S3. Summary of activity-related aspects of the engagement process that were identified by participants as needing improvement**.**

| **Aspect** | **Key issues** |
| --- | --- |
| Accountability | When things go wrong, patient partners often just leave a study with no exit interview or attempt to understand what happened. |
| Agency | The push to have patient partners engaged in all aspects of research sometimes leads to a loss of patient partner agency (i.e., patient partners must have a say in how/when they are engaged) and overlooking that patient engagement is context-specific. |
| Changes in group membership | It is unclear how to reconcile differences in patient partner input and ideas that result from patient partner turnover (due to, for example, the multi-year nature of research). |
| Depth and quality | Although there is a movement toward deep and integrated engagement approaches and recognition that patient engagement in research is not a “one size fits all” approach, heterogeneity in the depth and quality of engagement across studies and provinces remains. |
| Engagement liaisons | Studies do not consistently include a patient engagement liaison, and not everyone knows that you can “expense” and hire one. |
| Expectations | Oftentimes, patient partners do not know what to expect, do, or say when partnering – it is important to negotiate and establish expectations at the beginning of partnership. |
| Motivations | There is often limited time set aside for research partners to get to know each other and their collective motivations for engaging in research (including desired impacts), which diminishes the meaningfulness of engagement. |
| Power differentials | Power differentials are common among patient partners and academic researchers, and are associated with patient partners feeling that they lack genuine influence and prohibit their achieving their desired impact on a study. |
| Technology | Technology-related (i.e., access and knowledge) barriers disproportionately affect subgroups of the population that are underrepresented in patient engagement (e.g., individuals of low-income) and prevent patient partners from being able to partner to their full ability or at all. |
| Timing | Patient partners are not consistently engaged early in the research process (e.g., in the idea generation/grant planning stage), perhaps largely due to a lack of funding to support these early stages. |

Table S4. Overview of key studies examining future directions for patient engagement in research.

| **First Author/ Country/ Year/ Population** | **Summary of findings specific to future directions for patient engagement in research** |
| --- | --- |
| Bishop/  Canada/  2018/  Post-graduate trainees and early career investigators attending KT Canada Summer Institute 2017 | 2 main areas of focus for future directions:  (a) the knowledge base underlying patient engagement in research, including  - greater standardization of terminology in training programs  - clear guidance (e.g., resources, tools) to support patient engagement in research  - established evidence base to support, and improved evaluation of, patient engagement in research  (b) building and sustaining relationships, including  - relational aspects such as ensuring reciprocal relationships  - diversity and recruitment considerations  - increased reliance on established patient partner networks to help engage more patient partner and further establish patient engagement community |
| Vader/  Canada/  2022/  Trainees (undergraduate, postgraduate, fellows, health professions) engaged in pain research | 4 recommendations to improve patient engagement in research implementation:  (a) improved availability and accessibility of training opportunities and resources on patient engagement in research  (b) increased funding opportunities that support and/or require patient engagement in research  (c) creation of systems to support trainees in finding patient partners  (d) ensuring supervisors, departments, and institutions support and encourage patient engagement in research |
| Rouleau/  Canada/  2018/  Early career researchers (trainees and untenured researchers) holding select POR-specific scholarships, training and career awards | 3 key future features of patient-oriented research:  (a) crosses research disciplines  (b) taught in postgraduate programs  (c) has demonstrated and growing positive impacts  5 identified needs (to support future directions):  (a) multi-level (“micro, meso, and macro”) resources to ensure patient-oriented research’s sustainability and transition into the mainstream  (b) culture and paradigm changes  (c) time to establish the positive impacts of patient-oriented research  (d) integration of patient-oriented research into education curricula and programs  (e) addressing the fact that most current patient partners come with professional backgrounds  4 key roles for early career researchers in supporting the development and implementation of patient-oriented research:  (a) making a personal commitment to patient-oriented research  (b) developing personal skills that support effective engagement  (c) adopting reflexivity and critical reflection as part of one’s practices  (d) actively engaging in developing, raising awareness for, and disseminating the findings of patient-oriented research |
| PCORI/  USA/  2022/  Developed with “extensive stakeholder input” | Key aspects of PCORI’s 2023 Strategic Plan:  (a) A reaffirmed over-arching commitment to equity, diversity, and inclusion, and adaptability and responsiveness to an evolving healthcare and health research landscape through horizon scanning activities and regularly engaging with patients and stakeholders  (b) 5 stakeholder-identified areas of emphasis over the next 10 years:  - increasing awareness across the healthcare community about the value of patient-centered research  - diversity: strengthened emphasis on subpopulations in funded studies  - engagement processes: re-emphasized commitment to patient-centeredness, meaningful stakeholder engagement, and supporting ongoing development of partnerships across the healthcare  - comparative effectiveness research: enhanced focus on comparative effectiveness studies of both existing and new interventions, therapies, technologies, care delivery approaches, and healthcare policies  - expanding evidence synthesis capabilities: to meet the timely evidence needs of policymakers and payers  (c) 4 key elements that ensure a holistic approach to generating and promoting the use of evidence:  - funding patient-centered clinical effectiveness research that addresses questions important to patients and other stakeholders, and as guided by PCORI’s research agenda  - engagement of patients and other stakeholders across the continuum of PCORI’s work, as guided by the evaluation of the impact of different engagement practices on PCORI-funded research and PCORI’s guiding principles for engagement that help ensure equity and inclusion is an explicit goal of all research partnerships. Will also involve the continued expansion of resources designed to help train researchers and patients to build more effective research teams and broaden the community of individuals and groups  involved in health research and promoting early (e.g., proposal development stage) engagement that spans the lifecycle of funded activities.  - dissemination and implementation of research results that are relevant to needs and accessible for use in making informed decisions about health and health care  - investments to enhance the nation’s health research infrastructure, including in the science and methods of CER, the technical tools that can make research more efficient, and the workforce (through education) and delivery systems that are at the heart of patient care.  (d) 5 National priorities that represent PCORI’s long-term goals:  - increase evidence for existing interventions and emerging innovations in health  - enhance infrastructure to accelerate patient-centered outcomes research  - advance the science of dissemination, implementation, and health communication to enhance public understanding and uptake of research into practice  - expand stakeholder engagement, research, and dissemination approaches that lead toward achieving health equity in the United States  - accelerate progress toward an integrated learning health system |
| Staniszewska/  UK/  2018/  Key literature and national and international individuals and organizations representing a wide range of stakeholder groups | 3 key areas of future development for patient engagement in research in the NIHR:  (a) a perceived need to consolidate and use the available evidence to identify gaps in knowledge and continuous improvement of practice standards  (b) wider involvement of the general population in health research  (c) improved inclusion practices and greater diversity among patient partners, leaders, and mentors.  Future directions for the design and delivery of patient engagement in the NIHR:  (a) a shared aimed (and potentially central body) for patient engagement that underpins the complex network of organizations that comprise the NIH and informs the development of national policy supported by local practice,  (b) the development of flexible evidence-based methods to support engagement through evaluation of engagement practices, better identification of the key points where engagement has the greatest impact, and greater engagement of third sector representatives and community voluntary organizations as partners in the conduct of engagement and research  (c) collection of data to enable continuous improvement instead of only performance management, as potentially supported by National standards for patient engagement that can used to evaluate engagement quality and inform funding and ethical approval decisions.  2 key components of an overall vision for the future:  (a) patient engagement in research being a valued practice  (b) better evaluation of, and evidence to support, patient engagement that is embedded into regular practice. |

KT: Knowledge translation, NIHR: National Institute of Health and Care Research, PCORI: Patient-Centered Outcomes Research Institute, UK: United Kingdom, USA: United States of America.
